# Supplementary material for: Effect of family support and work resources in the relationship of economic constraints and work volition: Evidence from China
Source: PLoS One. 2025 Jan 17;20(1):e0310374. doi: 10.1371/journal.pone.0310374 (PMC11741595; doi:10.1371/journal.pone.0310374)
Supplement: Attachment — Submitted filename: Response to Reviewers.docx [file pone.0310374.s002.docx]

PONE-D-23-23939

Effect of Family Support and Work Resources in the Relationship of Economic Constraint and Work Volition : Evidence from China

PLOS ONE

Dear Dr. Henri Tilga

Thank you very much for the opportunity to revise our manuscript entitled “Effect of Family Support and Work Resources in the Relationship of Economic Constraint and Work Volition : Evidence from China”. We would like to thank you and the reviewers for their helpful comments. The manuscript has been carefully revised according to the comments raised by the reviewers. Please find a point-to-point response to reviewers’ comments below. Changes to the manuscript are highlighted in blue.

We believe that the revisions prompted by the reviewers’ comments have strengthened our manuscript. We hope it is now suitable for publication and look forward to hearing from you in course.

Yours faithfully

Academic Editor's Comments to Author:

1. **Please ensure that your manuscript meets PLOS ONE's style requirements, including those for** **file naming.**

**AUTHORS’ RESPONSE: Thank you very much for your review of our manuscript and very helpful comments. We have made revisions according to your comments. We have meet PLOS ONE's style requirements. As for the segment of “Abstract”, we have added the “Background” and “Contribution”. (pp. 2, marked in blue):**

**Abstract**

**Background**

As the pace of economic development slows, college students are facing an increasingly challenging employment landscape. For instance, the expansion of higher education has led to a swell in the number of job seekers, which has in turn intensified competition. Given the limited job opportunities, it's understandable that many college students are developing a pessimistic employment mindset. Therefore, it's crucial to explore how objective factors influence their work aspirations. But few studies have explored the role of mediating factors between the two, such as family and resource factors.Thus, this study examines the effects of family support and work resources between the relationship between economic constraints and work volition.

**Methods**

The study examined 1249 Chinese undergraduate students as participants ((714 men and 535 women; M_age_ = 19.32, SD = 1.50), using the questionnaire with the Wenjuanxing online survey tool. The questionnaire were collected between August, 2022 and December, 2022.

**Results**

The results showed that economic constraints have a significant negative impact on work volition via two mediators: family support and work resources. On the one hand, economic constraints negatively affect work volition through family support and work resource separately. On the other hand, economic constraints negatively predict family support and work resource, thus negatively impact work volition.

**Contribution**

The current study has established the independent mediating and chain-mediated effects of family support and work resources on the relationship between economic constraints and work volition. This deeper understanding of internal mechanisms provides valuable insights that can inform strategies for enhancing individual's work volition, particularly from the perspectives of economic constraints, family support, and work volition.

**Moreover, we have also corrected the format of the references. In the original article, we used superscripts in roman numerals. After we refer to the format of other articles published in the PlOSONE, we use the notation of Arabic numerals. For example, in the original text is was “family support refers to the positive resources that people acquired from their family relationships (Betz, 1989)^ⅲ^” But in the revised article, it is corrected “family support refers to the positive resources that people acquired from their family relationships[3].”**

**In addition, we revised file naming which is meet journal requirements, including “Response to Reviewers”, “ Revised Manuscript with Track Change Manuscript” and “Manuscript”.**

2. **Please provide additional details regarding participant consent. In the ethics statement in the Methods and online submission information, please ensure that you have specified what type you obtained (for instance, written or verbal, and if verbal, how it was documented and witnessed). If your study included minors, state whether you obtained consent from parents or guardians. If the need for consent was waived by the ethics committee, please include this information.**

**AUTHORS’ RESPONSE: Thank you for raising these important issues. To ensure the sample’s representativeness, the questionnaires of this study are respectively distributed to 1-2 cities of eastern, central, and western regions of China, including Chongqing, Shandong, Anhui, Guangxi, and Jiangsu provinces. After determining the target regions for questionnaire distribution, the research team provided detailed information about the questionnaire and shared the survey link with selected teachers in various provinces. These teachers were then tasked with distributing the questionnaires to students in their respective regions using WeChat, a widely utilized social media platform. This process was carried out between August 1st and December 31st, 2022. Informed consent was obtained from all subjects involved in the study by written style.** **The participants provided consent by completing the questionnaire (https://www.wjx.cn/vm/Q0VNs6G.aspx#) in Wenjuanxing. Note at the beginning of the questionnaire: This questionnaire is not mandatory, you have the right to withdraw at any time, if you fill out and submit the questionnaire, it is deemed that you understand and agree to our use of your relevant data. Therefore, every participant is informed. Their participation was anonymized. The study was conducted in accordance with the Declaration of Helsinki and approved by the Ethics Committee of the School of Education, Minzu University of China (approval date: June 1, 2022). (pp. 7-8, marked in blue):**

**Method**

**Participants**

To ensure the sample’s representativeness, the questionnaires of this study are respectively distributed to 1-2 cities of eastern, central, and western regions of China, including Chongqing, Shandong, Anhui, Guangxi, and Jiangsu provinces. Participants in the present study were 1249 undergraduate students with a mean age of 19.32 (*SD* = 1.50). There were 714 male (57.2%) and 535 female (42.8%) participants. Additionally, 598 (47.9%) were freshmen, 252 (20.2%) were sophomores, 206 (16.5%) were in their third year, and 193 (15.5%) were senior-year students. The participants were mainly from Chongqing, Shandong, Anhui, Guangxi, and Jiangsu provinces in China. Regarding monthly household income, participants reported 123 (9.8%), 301 (24.1%), 271 (21.7%), 193 (15.5%), 123 (9.8%), and 238 (19.1%), respectively, earned less than 2,000 RMB, 2,000–4,000 RMB, 4,000–6,000 RMB, 6,000–8000 RMB, 8,000–10,000 RMB, and more than 10,000 RMB.

**Procedure**

After determining the target regions for questionnaire distribution, the research team provided detailed information about the questionnaire and shared the survey link with selected teachers in various provinces. These teachers were then tasked with distributing the questionnaires to students in their respective regions using WeChat, a widely utilized social media platform. This process was carried out between August 1st and December 31st, 2022. Following data collection, the research team organized and conducted a comprehensive analysis of the collected data. Specifically, SPSS 21.0 was utilized to calculate measures such as skewness, kurtosis, internal consistency reliability, and common method bias (CMB). Meanwhile, AMOS 24.0 was used to compute average variance extracted (AVE), composite reliability (CR), heterotrait-monotrait ratio of correlations (HTMT), and the structural equation model (SEM). This analysis aimed to investigate the relationships among latent variables and assess the goodness of fit of the observed indicators on their associated latent constructs. Additionally, it evaluated all the hypothesized direct and indirect effects.

**Ethics statement**

Informed consent was obtained from all subjects involved in the study by written style. The participants provided consent by completing the questionnaire, and their participation was anonymized. The study was conducted in accordance with the Declaration of Helsinki and approved by the Ethics Committee of the School of Education, Minzu University of China (approval date: June 1, 2022).

3. **In your Data Availability statement, you have not specified where the minimal data set underlying the results described in your manuscript can be found. PLOS defines a study's minimal data set as the underlying data used to reach the conclusions drawn in the manuscript and any additional data required to replicate the reported study findings in their entirety. All PLOS journals require that the minimal data set be made fully available. For more information about our data policy, please see http://journals.plos.org/plosone/s/data-availability.**

**Upon re-submitting your revised manuscript, please upload your study’s minimal underlying data set as either Supporting Information files or to a stable, public repository and include the relevant URLs, DOIs, or accession numbers within your revised cover letter.**

**AUTHORS’ RESPONSE: Thank you for raising these important issues. We have uploaded our study’s minimal underlying data set as either Supporting Information files, which is named “data set”. The data that support this study are available from the corresponding author upon reasonable request.**

4. **PLOS requires an** **ORCID iD for the corresponding author in Editorial Manager on papers submitted after December 6th, 2016. Please ensure that you have an ORCID iD and that it is validated in Editorial Manager. To do this, go to ‘Update my Information’ (in the upper left-hand corner of the main menu), and click on the Fetch/Validate link next to the ORCID field.**

**AUTHORS’ RESPONSE: Thank you for raising these important issues. We have provided ORCID ID for the corresponding author in Editorial Manager on papers submitted.**

Reviewers’ Comments to Author:

**Reviewer: 1**

This paper is related to an interesting topic, namely Effect of Family Support and Work Resources in the Relationship of Economic Constraint and Work Volition : Evidence from China for PLOS ONE. However, the manuscript needs to be further developed in order to meet the expected academic requirements.

**AUTHORS’ RESPONSE: Thank you very much for your review of our manuscript and very helpful comments. We have made revisions according to your comments.**

**1.The introductory section of the document lacks the requisite depth and detail. It is essential to provide** **a more comprehensive introduction, one that not only sets the stage for the study but also explicitly identifies the** **existing knowledge gaps that this research aims to address. Without** **a clear articulation of these gaps in the introduction, the readers may find it challenging to understand the context and significance of the research.**

**AUTHORS’ RESPONSE: Thank you for raising this issue. We very much agree with your opinion and do find that the original article did not provide a comprehensive introduction regarding a clear articulation of existing knowledge gaps. We have added the following content according to your suggestion** **(pp. 3, marked in blue):**

Previous studies have examined the direct effects of economic constraints on work volition and the direct impact of family support and work resources on work volition. However, there are still knowledge gaps. For instance, previous research has not fully considered the mediating role of objective factors within the family and work in the relationship between family support and work volition, such as family support and work resources. Additionally, the internal mechanisms that economic constraints use to influence work volition through family support and work resources have not been fully explored.

Therefore, the goal of this study is to investigate the role played by family support and work resources in the relationship between economic constraints and work volition. We also aim to examine the complete pathway through which economic constraints indirectly affect work volition among Chinese college students facing employment issues.

**2.The segment dedicated to the** **literature review and** **hypotheses development exhibits some weaknesses.** **To bolster the quality of this section, it is imperative to delve into previous theories and research within the field.** **This entails a thorough discussion of the existing body of knowledge, highlighting key theories, findings, and their implications. Furthermore, it is crucial to elucidate the novel contribution of this research and provide a comprehensive explanation of how the model underpinning the study was constructed. The absence of this information leaves the model without a solid foundation, undermining the credibility and robustness of the research.**

**AUTHORS’ RESPONSE: Thank you for raising these important issues.**

(1) **As for the literature review and hypotheses development, in the original article, there are some very old literature and the the number of literature is also too low. Thus, for the segment of “Introduction” “Economic Constraints and Work Volition” “Economic Constraints and Work Volition” “Work resources as a second mediating variable” “Work resources as a second mediating variable”,we have increased the number of literature, and deleted some old ones and added many new ones in recent years, particularly 2021-2023. Moreover, we** **have delved into previous theories and researches, highlighting key theories, findings, and their implications** **(pp. 3-7, marked in blue):**

**Economic Constraints and Work Volition**

Work volition refers to an individual's sense of agency or freedom in making career choices [4]. Previous studies have examined the relationship between work volition and various variables, including economic constraints [17][18], academic satisfaction [6][19], social status [26][21], occupational engagement [20], social support [21], and others. Among these variables, economic constraints are considered to be the most significant.

Economic constraints refer to a lack of economic resources, which encompasses not only direct material goods but also access to economic resources [22][23]. Some studies conducted in different areas have found that individuals with greater economic constraints tend to have lower levels of career decision-making among university students and employees [7]. Tokar et al. (2018) and Cheung et al. (2020) examined different groups of subjects (workers and students) from different regions (United States and Hong Kong), and found that economic constraints were negatively related to work volition [24][25]. Zhang et al. (2019) conducted a study among Chinese college students and found that students from high-income families had higher work volition than those from low-income families. Allen et al. (2020) examined the relationship between economic constraints and work volition from a longitudinal perspective, which further proved that economic constraints negatively affect work volition [6].

**Family support as the first mediating variable**

Family support refers to the positive resources that individuals acquire from their family relationships [27], a dimension of social support that is a contextual factor known to influence career-related behavior. Many psychologists have emphasized the significance of family support in vocational development because it is directly linked to career search self-efficacy [28].Some studies have suggested that individuals supported by their families report greater work volition. For instance, Founad et al. (2015) developed the Family Influence Scale (FIS) to examine the influence of family on career and work choices. They also found that family's informational support and financial support can impact career and work choices, which can be seen as work volition [15]; in other words, family support can predict a higher level of work volition. Other studies have suggested that family support is significantly related to career optimism[11], which involves making free work choices with a positive mindset despite obstacles, fitting the definition of work volition. According to some studies conducted on Chinese college students, those with adequate career-related parental support are more likely to feel a sense of control, curiosity, confidence about work, and are able to choose the work that best suits their needs, interests, and values. In other words, individuals with greater family support will have greater work volition [29][30][31].

Furthermore, research indicates that there is a negative correlation between financial constraints and familial support. This implies that families who are struggling financially are less likely to provide their offspring with monetary assistance or various forms of financial aid. For instance, economic stress can negatively impact parenting styles, where high-quality parenting offers children comprehensive support. Studies have also shown that young individuals from families who are economically disadvantaged tend to encounter both internal and external issues[10], which can be attributed to the lack of familial support due to adverse economic circumstances.

Based on these studies, we establish the first hypothesis：

**H1:** Family support mediates the relationship between economic constraints and work volition.

**Work resources as a second mediating variable**

**Work resources** refer to the factors that stimulate personal growth, help achieve work goals, and mitigate the costs of job demands, which reflect physical, social, psychological, and organizational aspects[34]. They are intricately linked to experiences of job satisfaction, autonomy, purpose, engagement, meaningful work, and job performance[35]. According to Barbier et al. (2013), work and personal resources exert a causal effect on job engagement, which is a component of work volition[36]. Furthermore, individuals who lack essential work resources may feel burned out[37], making it more challenging to make informed job decisions, i.e., exhibiting a lower level of work volition. Cheung et al. (2020) examined the relationship between personal resources, constraints, and work volition among undergraduate students in the United States and Hong Kong. Their findings revealed a positive correlation between work resources and work volition, indicating that individuals with greater work resources are more likely to possess the capacity to make informed work decisions[24]. Choi et al. (2022) examined the relationship between social class and work volition, and their findings revealed a positive correlation between the two[38]. This supports the theory that individuals from lower social classes, who may face economic constraints, tend to exhibit lower levels of work volition. A study among Chinese college students revealed that those from high socioeconomic backgrounds, who have greater access to helpful work resources, tend to exhibit higher levels of work volition [39]. Additionally, research has shown that economic constraints can limit individuals' access to work resources. Bourdieu (2008) suggested that economic constraints limit individuals' access to these resources, as individuals or their families may need to pay for them, which can be difficult when facing economic constraints [40]. Duffy et al. (2016) further suggested that economic constraints limit capital resources that can be applied in the workplace, making it more difficult for individuals in economic constraints to acquire the resources that privileged individuals take for granted [17]. A study among labour market entrants found that individuals whose parents had higher education levels, were employed, or were from the service class (indicating greater economic income) had greater access to job-finding resources [41]. In other words, less economic constraint promotes greater access to job resources.

Based on these studies, we establish the second hypothesis：

**H2:** Work resources mediate the relationship between economic constraints and work volition.

**Family support and Work resources**

About the relationship between these two mediated variables, some studies indicated the relationship between these two mediating variables has been explored in several studies, which suggest that family support is associated with work resources. Bourdieu's theory (2008) postulates that families with diverse capital can provide individuals with resources, including work-related resources such as a professional network, job opportunities, and a clear understanding of job rules [40]. Researchers have also conducted empirical studies to test this relationship. Fass et al. (2013) found that families with high occupational status can provide their children with work resources such as professional training, communication skills, and managerial abilities [43]. In other words, individuals with various forms of family support are likely to have access to ample work resources. Wayne and Matthews et al. (2020) conducted a cross-sectional survey of employees and found that work resources are associated with lower work-to-family conflict[44], which suggests that individuals can receive greater family support for their work. Furthermore, a study focusing on college students revealed that higher levels of family cohesion were associated with career thoughts, an important aspect of work volition. This suggests that a harmonious family can provide greater support and promote a higher level of work volition[45].

Based on these studies and combine the findings that family support and work resource play important roles between economic constraints and work volition, we established the third hypothesis:

H3: Family support and work resources played a chain-mediating role in the relationship between economic constraints and work volition.

(2) **As for the novel contribution, in the original article, we only provided practical contributions, thus, we have added the theoretical contributions in the segment of “Cnotribution”(pp.12, marked in blue).**

**In the Contribution**, we have added: The results of this study offer valuable insights into the roles played by family support and work resources in the processes linking economic strain to work volition. Specifically, having adequate family support and work resources can enhance the work volition of individuals facing economic difficulties. Therefore, our findings suggest potential avenues for targeted interventions among college students facing economic constraints, with the aim of boosting their work volition and promoting more effective career development.

(3) **As for the comprehensive explanation of how the model underpinning the study was constructed.**

**AUTHORS’ RESPONSE: Thank you for raising these important issues. We have added the conceptual model of family support and work resources as mediators and social of the relation between the economic constraints and work volition.**

3. **The summary or conclusion of the study appears to be overly succinct and leaves some key elements unaddressed.** **A comprehensive conclusion should not only summarize the key findings but also provide answers to the research questions posed at the outset of the study. By failing to address these questions in the conclusion, the research may be perceived as incomplete and lacking in closure. A more comprehensive summary and conclusion would help readers to better grasp the implications and significance of the study's findings.**

**AUTHORS’ RESPONSE: Thank you for raising these important issues. We have added new content about answers to the research questions posed at the end of introduction, that is: we aim to explore what role family support and work resources play between economic constraints and work volition, and examine the complete pathways through which the economic constrains indirectly affects work volition among Chinese college students who faced employment issues. Therefore, the answers has been provided in the “Conclusion”****(pp.14, marked in blue) .**

The present study, which focused on Chinese college students, delved into the relationship between economic constraints and work volition, taking into account the mediating effects of family support and work resources. The findings confirmed the mediating roles and chain mediating roles of these factors in the relationship. Specifically, three indirect pathways were identified: the economic constraints affecting work volition through family support, through work resources, and through a combined effect of family support and work resources. While acknowledging several limitations, the study provides valuable directions for future research on the importance of family support and work resources in career development. It also underscores the need for schools, families, and society to recognize and act on these factors to promote positive career outcomes for students.

**Reviewer: 2**

**Thank you for submitting your paper "Effect of Family Support and Work Resources in the Relationship of Economic Constraint and Work Volition : Evidence from China" to this journal "Plos One". I read your paper and gave my concern as follows:**

**AUTHORS’ RESPONSE: Thank you very much for your review of our manuscript and very helpful comments. We have made revisions according to your comments.**

**1.The abstract is not properly written. The background, and contributions of the paper must be included.**

**AUTHORS’ RESPONSE: Thank you for raising this issue.We have provided the background and contributions in the “Abstract” (pp.2, marked in blue).**

**Background**

As the pace of economic development slows, college students are facing an increasingly challenging employment landscape. For instance, the expansion of higher education has led to a swell in the number of job seekers, which has in turn intensified competition. Given the limited job opportunities, it's understandable that many college students are developing a pessimistic employment mindset. Therefore, it's crucial to explore how objective factors influence their work aspirations. But few studies have explored the role of mediating factors between the two, such as family and resource factors.Thus, this study examines the effects of family support and work resources between the relationship between economic constraints and work volition.

**Contribution**

The current study has established the independent mediating and chain-mediated effects of family support and work resources on the relationship between economic constraints and work volition. This deeper understanding of internal mechanisms provides valuable insights that can inform strategies for enhancing individual's work volition, particularly from the perspectives of economic constraints, family support, and work volition.

**2.Introduction section lacks the rationale of writing this paper. The paper must craft the research problem and essence of the problem to be addressed. Moreover, the contributions of the authors must be justified through a solid research gap that has not yet addressed.**

**AUTHORS’ RESPONSE: Thank you for raising this issue. The rationale of writing this paper includes two aspects. On the one hand, the Chinese youth group is facing increasing difficulties in finding employment, we want to explore what can be done to facilitate their professional development. On the other hand, there is a research gap that has not addressed. Few studies has yet explored the internal mechanisms by which the economic constrains influence work volition, in particular the specific roles of family support and work resources in the relationship between the economic constrains and work volition. Thus, we have added these specific content into the “Introduction” (pp.3, marked in blue).**

In recent years, due to the economic downturn and the college expansion plan, the youth demographic, primarily comprised of university graduates, is encountering increasingly greater challenges in securing gainful employment[1]. Amidst such immense pressure, it is noteworthy that a strong work ethic can serve as a valuable asset in securing suitable employment opportunities in the future.[2][3].

Previous studies have examined the direct effects of economic constraints on work volition and the direct impact of family support and work resources on work volition. However, there are still knowledge gaps. For instance, previous research has not fully considered the mediating role of objective factors within the family and work in the relationship between family support and work volition, such as family support and work resources. Additionally, the internal mechanisms that economic constraints use to influence work volition through family support and work resources have not been fully explored.

**3.Literature review of the paper or the developing of the hypothesis was based on very old literature. The author must** **develop or justify those relationships based on recent papers/citations.**

**AUTHORS’ RESPONSE: Thank you for raising this issue. Some very old literature of the literature review and the developing of the hypothesis has been removed, for example, those** **written by Xin, 2005; Betz, 1989; Nota et al., 2007; Dietrich and Kracke, 2009; Lee et al., 2009. In addition, we have cited some new literature written in recent years. By reviewing this literature in recent years, we justified the relationship between family support, work resources, economic constraints, work volition. Specifically, economic constrains negatively affect family support (Wang and Li et al., 2022; Sprung, 2022). Family support plays a significant role of individual’ career development (Newman and Dunwoodie et al., 2022; Agrawal and Singh, 2022; Mehreen and Ali, 2022; Yang and Fang et al., 2023; Wang and Zhang et al., 2023; Zeng and Li et al,2023; Zhang and Yuen et al., 2021;Song and Mu et al., 2022). Work resources are also regarded as one of the necessary resources for individuals in their vocational development (Pursio and Siukola et al.,2021; Cheung et al., 2020 ). However, some of the very classic literature are still preserved, especially those that define the meaning of key variables. For example, Betz provided the definition of family support in 1989, and Brief, Konovsky, Goodwin and Link offered the definition of economic constraints in 1995. Moreover, some views in the theory raised by Bourdieu in 2008 provide essential theoretical support for our research, thus, they are also retained.**

4. **In methodology, the authors did not ground this paper. Particularly, the research design, sampling design, sampling frame, data collection procedure, etc were not sufficiently explained.**

**AUTHORS’ RESPONSE: Thank you for raising this issue. In the segment of “Method”, we have added relevant information about the research design, sampling design, sampling frame, data collection procedure and provided sufficient explanation of these (pp.7-8, marked in blue).**

**Method**

**Participants**

To ensure the sample’s representativeness, the questionnaires of this study are respectively distributed to 1-2 cities of eastern, central, and western regions of China, including Chongqing, Shandong, Anhui, Guangxi, and Jiangsu provinces. Participants in the present study were 1249 undergraduate students with a mean age of 19.32 (*SD* = 1.50). There were 714 male (57.2%) and 535 female (42.8%) participants. Additionally, 598 (47.9%) were freshmen, 252 (20.2%) were sophomores, 206 (16.5%) were in their third year, and 193 (15.5%) were senior-year students. The participants were mainly from Chongqing, Shandong, Anhui, Guangxi, and Jiangsu provinces in China. Regarding monthly household income, participants reported 123 (9.8%), 301 (24.1%), 271 (21.7%), 193 (15.5%), 123 (9.8%), and 238 (19.1%), respectively, earned less than 2,000 RMB, 2,000–4,000 RMB, 4,000–6,000 RMB, 6,000–8000 RMB, 8,000–10,000 RMB, and more than 10,000 RMB.

**Procedure**

After determining the target regions for questionnaire distribution, the research team provided detailed information about the questionnaire and shared the survey link with selected teachers in various provinces. These teachers were then tasked with distributing the questionnaires to students in their respective regions using WeChat, a widely utilized social media platform. This process was carried out between August 1st and December 31st, 2022. Following data collection, the research team organized and conducted a comprehensive analysis of the collected data. Specifically, SPSS 21.0 was utilized to calculate measures such as skewness, kurtosis, internal consistency reliability, and common method bias (CMB). Meanwhile, AMOS 24.0 was used to compute average variance extracted (AVE), composite reliability (CR), heterotrait-monotrait ratio of correlations (HTMT), and the structural equation model (SEM). This analysis aimed to investigate the relationships among latent variables and assess the goodness of fit of the observed indicators on their associated latent constructs. Additionally, it evaluated all the hypothesized direct and indirect effects.

5. **Measurement model was not supported with the required estimates. Particularly, I don't observed AVE, CR, and HTMT issues in this paper.**

**AUTHORS’ RESPONSE: Thank you for raising this issue.**

**(1)****In the Procedure of original text, we wrote about the measurement process in this way: data for the current study were collected from undergraduate students using the Wenjuanxing online survey tool (https://www.wjx.cn/vm/tUuoLX) between August 1 and December 31, 2022. It took up to 15 minutes to complete the survey. It did not cover AVE, CR, and HTMT issues. Therefore, we have analyzed the collected data again and added measurement process and results of AVE, CR, and HTMT issues in this research, which is shown in the segment of Procedure . (pp.8, marked in blue).**

**Procedure**

After determining the target regions for questionnaire distribution, the research team provided detailed information about the questionnaire and shared the survey link with selected teachers in various provinces. These teachers were then tasked with distributing the questionnaires to students in their respective regions using WeChat, a widely utilized social media platform. This process was carried out between August 1st and December 31st, 2022. Following data collection, the research team organized and conducted a comprehensive analysis of the collected data. Specifically, SPSS 21.0 was utilized to calculate measures such as skewness, kurtosis, internal consistency reliability, and common method bias (CMB). Meanwhile, AMOS 24.0 was used to compute average variance extracted (AVE), composite reliability (CR), heterotrait-monotrait ratio of correlations (HTMT), and the structural equation model (SEM). This analysis aimed to investigate the relationships among latent variables and assess the goodness of fit of the observed indicators on their associated latent constructs. Additionally, it evaluated all the hypothesized direct and indirect effects.

**In the Results of original text, we wrote about the preliminary analyses in this way: In the Procedure of original text, we wrote about the measurement process in this way: Before analyzing the data, we conducted preliminary analyses using IBM SPSS 21.0. The skewness and kurtosis of all variables were -0.66 and -0.78, respectively. On account of the absolute values of skewness ≤ 3.0 and kurtosis ≤ 10.0 (Weston and Gore, 2006), the shape of the data distribution in the current study may not be severely non-normal. .It did not cover AVE, CR, and HTMT issues. Therefore, we have analyzed the collected data again and added measurement process and results of AVE, CR, and HTMT issues in this research, which is shown in the segment of Procedure . (pp.9-10, marked in blue).**

**Preliminary Analyses**

Before analyzing the data, we conducted preliminary analyses using IBM SPSS 21.0. The skewness and kurtosis of all variables were -0.66 and -0.78, respectively. On account of the absolute values of skewness ≤ 3.0 and kurtosis ≤ 10.0[55], the shape of the data distribution in the current study may not be severely non-normal. All items in the questionnaire were subjected to Harman’s single-factor test, and the results showed that the first factor explained 33.49% of the total variance, which is below the critical value of 40%. This indicates that there is no significant presence of common method bias.

Moreover, the results showed that the CR of the four scales were 0.95, 0.87, 0.90 and 0.83 respectively, denoting high composite reliability based on the principle of Hair et al.(1998)[56]. The results showed that the AVE of the four scales were 0.79, 0.63, 0.75 and 0.42, and the square root of the number AVE on the diagonal of the scale mostly higher than the Pearson correlation among scales, indicating that these questionnaire have strong convergent validity and discriminant validity based on the principle of Fornell and Larcker（1981）[45]. And, the results showed that the HTMT between the four scales were less than 0.85, further denoting high composite reliability based on the principle of Henseler, Ringle, & Sarstedt(2015)[58]. The descriptive statistics also are presented (see Table 1).

**(2)Moreover, after analyzing again, some data of measurement model and structural model changed. We have made changes in the segment of “Measurement model” “Structural model” and “Discussion” (pp.8-10, marked in blue).**

**Measurement Model**

We constructed measurement models to examine correlations among latent variables and the goodness of fit of the observed indicators on their associated latent constructs with Amos Graphics 24.0; we found χ^2^/df=4.64, RMSEA = 0.05, TLI = 0.96, CFI = 0.97, and SRMR = 0.04, indicating a good fit according to some thresholds^[1]^. We, then, tested these structural models. The factor correlations and descriptive statistics are presented in Table 1.

**Structural Model**

We tested structural models that contained all the hypothesized direct and indirect effects and found that most paths were significant. The results of *χ*^2^/df=4.64, RMSEA = 0.05, TLI = 0.96, CFI = 0.97, and SRMR = 0.04 indicate a good fit according to some thresholds^[2]^. In terms of direct paths, economic constraints failed to predict work volition directly (β = -0.04, SE = 0.03, p=0.25, 95% CI [-0.09, 0.03]). Economic constraints had significant direct effects on family support (β = -0.21, SE = 0.03, p <0.001, 95% CI [ -0.27, -0.14]) and work resources (β = -0.11, SE = 0.03, p <0.01, 95% CI [-0.18, -0.05]); family support had significant direct effects on work resources (β = 0.47, SE = 0.03, p <0.001, 95% CI [0.40, 0.53]) and work volition (β = 0.16, SE = 0.04, p <0.001, 95% CI [0.08, 0.23]); work resources had significant direct effects on work volition (β = 0.47, SE = 0.03, p <0.001, 95% CI [0.40, 0.53] and β = 0.53, SE = 0.04, p <0.001, 95% CI [0.46, 0.60], respectively).

As for the indirect paths, economic constraints had a significant indirect effect on work volition via family support and work resources (β = -0.01, SE = 0.004, p <0.001, 95% CI [-0.02, -0.01] and β = -0.02, SE = 0.01, p <0.01, 95% CI [-0.04, -0.01], respectively). Additionally, we examined a chain mediation that economic constraints to work volition via family support and work resources (β = -0.02, SE = 0.004, p <0.001, 95% CI [-0.03, -0.01]). All paths are listed in Tables 2 and 3.

**Discussion**

This study aims to test the effect of family support and work resources on the relationship between economic constraints and work volition using the data collected from college students in China. Overall, the results confirmed the hypotheses that economic constraints have a significant negative impact on work volition *via* two psychological mediators: family support and work resources.

Regarding the first hypothesis, economic constraints negatively affected work volition through family support and the ratio of indirect effcet is 0.49, which means family support plays an important meditating role. Specifically, those who suffered economic hardships received less family support, resulting in a lower level of ability to make career-related decisions, which is consistent with the findings of previous studies. For example, it is showed that students with economic constraints are more likely to face a variety of problems, which is not conducive for work volition [10]. Kim et al. (2016) also found that those who felt higher family support had more work volition than those who did not. The conclusions can be explained by the following reasons[51]. First, for students with financial difficulties, their parents spend most of their time working hard to earn money to meet their needs for necessary expenses; thus, these families do not have extra time or energy to provide abundant various support, both physically and emotionally. Second, people with more family support have more stress resistance[47]; therefore, they have enough confidence and courage to make the best career choices without being afraid to take risks of failure.

Regarding the second hypothesis, economic constraints significantly predict work volition through work resources, and the ratio of indirect effcet is 0.63, which means work resources have a significant meditating effect. To elaborate, students in bad economic conditions are less likely to have access to ample work resources, and they will feel less freedom in their career choice. This result is consistent with the theory proposed by Bourdieu (2008)[50], which indicates that among the three guises of capital existing in the world (i.e., economic, cultural, and social), both cultural and social capitals can be derived from economic capital. This implies that economic constraints limit the gain of other types of capital, particularly work resources, in our case. Moreover, it is also in line with the Conversation of Resources theory[48], the core of stress is the fear of loss of resources; that is, people with more work resources are more likely to resist various work stresses. Therefore, they can perceive the freedom to choose a career regardless of any potential stress or challenge. The findings can be attributed some explanations. First, the primary reason may be that most access to career resources, such as taking job training classes or consulting with job agencies, requires payment; however, people with economic difficulties do not have enough money to bear these costs. Second, students with abundant work resources usually acquire the professional knowledge and skills required for their work. These students always have a clear career plan that can partially increase their level of work volition.

Regarding the third hypothesis, family support and work resources act as a chain intermediary between economic constraints and work volition, and the ratio of indirect effcet is 0.60, which means both family support and work resources have important meditating effect. Specifically, economic constraints negatively predict family support. Family support has a significant positive effect on work resources, and work resources also have a significant positive effect on work volition. The relationships between economic constraints and family support and work resources and volition have been discussed above; here, we have discussed the relationship between family support and work resources. This finding indicates that people with adequate family support acquire better work resources, which is consistent with the findings of previous studies. It was found that interpersonal network is very important to people’s life and career because it will bring valuable information which can not be acquired through one's own efforts[49]. For college students, most of their related-work interpersonal network is supported by their family. Thus, with the more family support, they are more likely to possess further information, such as vocational information, employment opportunities, and necessary encouragement, which can bring them better work resources. In sum, combined with the theory raised by Bourdieu (2008) that families in an economic dilemma are difficult to receive sufficient support[50], it is confirmed that those who have higher economic constrains perceive less family support, and lacking family support further limits the gain of work resource and leads to lower work volition.

**(3)Some date in tables have also been revised** **(pp.14-15, marked in blue).**

**Table 1 Descriptive Statistics and Correlations of Variables**

|  | CR | AVE | 1 | 2 | 3 | 4 |
| --- | --- | --- | --- | --- | --- | --- |
| 1.Economic constraints | 0.95 | 0.79 | 0.89 | 0.24 | 0.22 | 0.14 |
| 2. Family Support | 0.87 | 0.63 | -0.22** | 0.80 | 0.51 | 0.44 |
| 3. Work Resources | 0.90 | 0.75 | -0.21** | 0.45** | 0.87 | 0.61 |
| 4. Work Volition | 0.83 | 0.42 | -0.43** | 0.37** | 0.44** | 0.65 |
| ***M*** | | | 3.78 | 5.14 | 3.50 | 4.51 |
| ***SD*** | | | 1.50 | 1.15 | 0.76 | 0.73 |

Note: ^*^*p* < 0.05, ^**^*p* < 0.01, ^***^*p* < 0.001. The data on the diagonal of different questionnaires represent the square root of AVE. The data below the diagonal represent the correlation coefficients between different questionnaires. The data above the diagonal represent the HTMT values of the questionnaires.

**Table 2 Paths Coefficient from Structural Model**

| **Paths** | **β** | **SE** | **95% confidence interval**  **bootstrap bias corrected** | |
| --- | --- | --- | --- | --- |
|  |  |  | **Lower bound** | **Upper bound** |
| EC→FS | -0.21^***^ | 0.03 | -0.27 | -0.14 |
| EC→WR | -0.11^**^ | 0.03 | -0.18 | -0.05 |
| EC→WV | -0.04 | 0.03 | -0.09 | 0.03 |
| FS→WR | 0.47^***^ | 0.03 | 0.40 | 0.53 |
| FS→WV | 0.16^***^ | 0.04 | 0.08 | 0.23 |
| WR→WV | 0.53^***^ | 0.04 | 0.46 | 0.60 |

Note: EC=economic constraints; FS=family support; WR=work resources; WV=work volition. ^*^*p* < 0.05, ^**^*p* < 0.01, ^***^*p* < 0.001

**Table 3 Direct and Indirect Effects from the Structural Model**

| **paths** | **effect type** | **β** | **SE** | **95% confidence interval bootstrap bias corrected** | | **ratio** |
| --- | --- | --- | --- | --- | --- | --- |
|  |  |  |  | **Lower bound** | **Upper bound** |  |
| EC→FS/WR  /FS&WR→WV | Direct effect | -0.01 | 0.01 | -0.04 | 0.01 | 0.51/0.37  /0.40 |
| EC→FS→WV | Total effcet | -0.03^*^ | 0.01 | -0.05 | -0.004 | - |
|  | Indirect effcet | -0.01^***^ | 0.004 | -0.02 | -0.01 | 0.49 |
| EC→WR→WV | Total effcet | -0.04^**^ | 0.01 | -0.07 | -0.01 | - |
|  | Indirect effcet | -0.02^**^ | 0.01 | -0.04 | -0.01 | 0.63 |
| EC→FS→WR→WV | Total effcet | -0.03^*^ | 0.004 | -0.06 | -0.01 | - |
|  | Indirect effcet | -0.02^***^ | 0.01 | -0.03 | -0.01 | 0.60 |

Note: EC=economic constraints; FS=family support; WR=work resources; WV=work volition. ^*^*p* < 0.05, ^**^*p* < 0.01, ^***^*p* < 0.001

6. **Where is** **CMB reporting.**

**AUTHORS’ RESPONSE: Thank you for raising this issue. During the process of date analysis, CMB was used to calculate the internal consistency. The results of the calculation are presented in the segment of “Result”. All items in the questionnaire were subjected to Harman’s single-factor test, and the results showed that the first factor explained 33.49% of the total variance, which is below the critical value of 40%. This indicates that there is no significant presence of common method bias (pp.9, marked in blue).**

Before analyzing the data, we conducted preliminary analyses using IBM SPSS 21.0. The skewness and kurtosis of all variables were -0.66 and -0.78, respectively. On account of the absolute values of skewness ≤ 3.0 and kurtosis ≤ 10.0[55], the shape of the data distribution in the current study may not be severely non-normal. All items in the questionnaire were subjected to Harman’s single-factor test, and the results showed that the first factor explained 33.49% of the total variance, which is below the critical value of 40%. This indicates that there is no significant presence of common method bias.

7. **A separate section on** **the strengths or contributions must be added after discussion.**

**AUTHORS’ RESPONSE: Thank you for raising this issue. After discussion, we have provided the contributions and added some information of theoretical and practical contributions (pp.12-13, marked in blue).**

**Contribution**

The results of this study offer valuable insights into the roles played by family support and work resources in the processes linking economic strain to work volition. Specifically, having adequate family support and work resources can enhance the work volition of individuals facing economic difficulties. Therefore, our findings suggest potential avenues for targeted interventions among college students facing economic constraints, with the aim of boosting their work volition and promoting more effective career development.

First, based on the finding that limited economic conditions directly undermine the level of family support and work resources and work volition of university students. Governments and colleges are encouraged to establish specialized scholarships for those who are constrained by economic burden; thus, they will spend more time considering future career plans instead of worrying about their livelihood. In addition, vocational training and educational institutions are expected to offer free or discounted courses for students to receive vocational certificates, which could provide them with better career options. Moreover, recruitment companies should set up more paid internship opportunities for undergraduates in order to enrich their work resources. Therefore, they will be more experienced when making work choices.

Second, our findings suggest that family support is a salient factor that affects work resources and work volition. [Therefore](javascript:;), more active strategies are required to promote family support. Parents are ought to provide their children with substantial support; for example, they can give their children funds to take part in lessons in order to get internship opportunities to improve their work-related beliefs and actual abilities. Families can also offer emotional support. Specifically, parents should try to understand and respect their children’s personal career choices, democratically raise appropriate suggestions, and encourage their children to explore their future without burden.

Finally, the mediating effects of work resources can inform appropriate interventions. Universities or vocational organizations are supported to disseminate considerable career-related information, such as development trends and hotspots of various professions. More experiential training and internships should also be offered, allowing students to gain experience and master skills matching their future work, which will increase their confidence in making work choices.

This study adds to the literature of the relationship between economic constrains and work volition by validating family support and work resource as novel mediating roles. Under the realistic background of college students’ difficult employment, future research is required to further explore the internal mechanism of economic influence on work volition. We believe that the data from the present study could provide future research useful insights into understanding specific impacts at the family support and resource level.

**8.Notably, the authors ignored recent papers to cite in their paper. I will ask them to search and cite papers from recent years (particularly, 2021-2023).**

**AUTHORS’ RESPONSE: Thank you for raising this issue. We have cited papers from recent years in 2021-2023. Particularly in the segment of “Introduction” and “Discussion”.** **In the original article, there were 44 papers were cited in total, including 6 in 2021-2023, accounting for 13.6%. After our revising, there are 60 cited papers, particularly, including 19 in 2021-2023, accounting for 31.6 %, increasing 18.0%（following text in blue）.**

**（1）In the “Introduction”, we added new cited papers in recent years**: In addition, it was also found that economic constrains could intensify conflicts and distress between family members (Wang and Li et al., 2022; Sprung, 2022), which is not conducive for adolescents getting family support. Meanwhile, studies suggested that economic constrains was related to work resources (Conger et al., 2022). For the Chinese who have a tradition of attaching importance to family values, family support plays a significant role during individual’s whole growth, including career development (Newman and Dunwoodie et al., 2022; Agrawal and Singh, 2022; Mehreen and Ali, 2022; Yang and Fang et al., 2023; Wang and Zhang et al., 2023). Moreover, work resources are also regarded as one of the necessary resources for individuals in their vocational development (Pursio and Siukola et al., 2021).

**（2）In the “Family support as the first mediating variable”, we added new cited papers in recent years:** For example, Wang and Zhang (2023) suggested that adolescences’ who perceive more family support show more career engagement; that is, can predict higher level of work volition. Studies have also suggested that family support was significantly related to career optimism(Newman and Dunwoodie et al., 2022), meaning with an positive mindset to making free work choice despite obstacles, which fits the definition of work volition. According to some studies ,conducted on Chinese college students, those with sufficient career-related parental support are more likely to feel concern, control, curiosity, and confidence of work, can choose the work best suiting their needs, interests, and values. In other words, people with higher family support will have more work volition (Zeng and Li et al,2023; Zhang and Yuen et al., 2021;Song and Mu et al., 2022).

In addition, studies suggest that a negative relationship exists between economic constraints and family support, which means families in economic constraints are less likely to support their offspring with money or other forms of financial help. For example, economic strain negatively affects the way of parenting (Conrad and Paschall et al., 2019; Nuri et al., 2019), a high-quality parenting will offer children comprehensive support.

**（3）****In the “Work resources as a second mediating variable”, we added new cited papers in recent years:** These aspects relate to experiences of job satisfaction, autonomy, purpose, engagement, and meaningful work, and job performance (Janse van Rensburg et al., 2018).Cheung et al. (2020) have proved that work resources are positively associated with work volition. Alix (2017) indicated that individuals should have work resources such as social connections and assets, to support themselves, which can be used to build work volition in poor circumstances. Barbier et al. (2013) suggested that work and personal resources have a causal effect on job engagement, which can be seen as part of work volition. In addition, It was suggested that when individual fails to be provided essential work resource, he will feel burned out (Takawira et al.,2014), which may lead to lower level of work volition.

**（4）In the “Introduction”, we added new cited papers in recent years:** For example, it is showed that students with economic constraints are more likely to face a variety of problems, which is not conducivefor work volition (Conger et al., 2022). Second, people with more family support have more stress resistance (Liu; Diao; and Liu et al.,2022 ). It was found that interpersonal network is very important for people’s life and career because it will bring some valuable information which can not be acquired by one's own efforts(Lu.,2021).
