# Supplementary material for: Effect of family support and work resources in the relationship of economic constraints and work volition: Evidence from China
Source: PLoS One. 2025 Jan 17;20(1):e0310374. doi: 10.1371/journal.pone.0310374 (PMC11741595; doi:10.1371/journal.pone.0310374)
Supplement: Attachment — Submitted filename: Response to Reviewers.docx [file pone.0310374.s003.docx]

PONE-D-23-23939

Effect of Family Support and Work Resources in the Relationship of Economic Constraints and Work Volition : Evidence from China

PLOS ONE

Dear Dr. Henri Tilga

Thank you very much for the opportunity to revise our manuscript entitled “Effect of Family Support and Work Resources in the Relationship of Economic Constraint and Work Volition : Evidence from China”. We would like to thank you and the reviewers for their helpful comments. The manuscript has been carefully revised according to the comments raised by the reviewers. Please find a point-to-point response to reviewers’ comments below. Changes to the manuscript are highlighted in blue.

We believe that the revisions prompted by the reviewers’ comments have strengthened our manuscript. We hope it is now suitable for publication and look forward to hearing from you in course.

Yours faithfully

Reviewers’ Comments to Author:

**Reviewer: 1**

**1.While the article holds certain research value, it falls short in delivering compelling theoretical discussions. A well-developed theoretical foundation is crucial for any academic work. Moreover, the paper tends to make numerous assumptions and addresses a wide array of research questions, lacking the necessary focus. It is suggested that the author refines the research questions for a more coherent presentation. In all, the current manuscript is not well-grounded in the related literature, nor does it develop a theoretical framework that is novel enough for the journal readers.**

**AUTHORS’ RESPONSE: Thank you very much for your review of our manuscript and very helpful comments. We have made revisions according to your comments. We have added more relevant research and viewpoints to make the theoretical foundation more profound.** **(pp. 4-7, marked in blue):**

**Family support as the first mediating variable**

Family support refers to the positive resources that individuals acquire from their family relationships [27], a dimension of social support that is a contextual factor known to influence career-related behavior. Many psychologists have emphasized the significance of family support in vocational development because it is directly linked to career search self-efficacy [28]. On the one hand, family support can bring emotional encouragement and work support to individuals. On the other hand, it can relieve personal stress so that they can have more energy to devote to their work [61]. Some studies have suggested that individuals supported by their families report greater work volition. For instance, Founad et al. (2015) developed the Family Influence Scale (FIS) to examine the influence of family on career and work choices. They also found that family's informational support and financial support can impact career and work choices, which can be seen as work volition [15]; in other words, family support can predict a higher level of work volition. Other studies have suggested that family support is significantly related to career optimism[11], which involves making free work choices with a positive mindset despite obstacles, fitting the definition of work volition. According to some studies conducted on Chinese college students, those with adequate career-related parental support are more likely to feel a sense of control, curiosity, confidence about work, and are able to choose the work that best suits their needs, interests, and values. In other words, individuals with greater family support will have greater work volition [29][30][31].

Furthermore, research indicates that there is a negative correlation between financial constraints and familial support. This implies that families who are struggling financially are less likely to provide their offspring with monetary assistance or various forms of financial aid. For instance, economic stress can negatively impact parenting styles, where high-quality parenting offers children comprehensive support. Studies have also shown that young individuals from families who are economically disadvantaged tend to encounter both internal and external issues[10], which can be attributed to the lack of familial support due to adverse economic circumstances.

Based on these studies, we establish the first hypothesis：

**H1:** Family support mediates the relationship between economic constraints and work volition.

**Work resources as a second mediating variable**

**Work resources** refer to the factors that stimulate personal growth, help achieve work goals, and mitigate the costs of job demands, which reflect physical, social, psychological, and organizational aspects[34]. They are intricately linked to experiences of job satisfaction, autonomy, purpose, engagement, meaningful work, and job performance[35]. Numerous empirical studies have found that when individuals lose resources at work, they are more likely to have negative physiological outcomes such as burnout and depression[62], which could adversely affects the work volition. According to Barbier et al. (2013), work and personal resources exert a causal effect on job engagement, which is a component of work volition[36]. Furthermore, individuals who lack essential work resources may feel burned out[37], making it more challenging to make informed job decisions, i.e., exhibiting a lower level of work volition. Cheung et al. (2020) examined the relationship between personal resources, constraints, and work volition among undergraduate students in the United States and Hong Kong. Their findings revealed a positive correlation between work resources and work volition, indicating that individuals with greater work resources are more likely to possess the capacity to make informed work decisions[24]. Choi et al. (2022) examined the relationship between social class and work volition, and their findings revealed a positive correlation between the two[38]. This supports the theory that individuals from lower social classes, who may face economic constraints, tend to exhibit lower levels of work volition. A study among Chinese college students revealed that those from high socioeconomic backgrounds, who have greater access to helpful work resources, tend to exhibit higher levels of work volition [39]. Additionally, research has shown that economic constraints can limit individuals' access to work resources. Bourdieu (2008) suggested that economic constraints limit individuals' access to these resources, as individuals or their families may need to pay for them, which can be difficult when facing economic constraints [40]. Duffy et al. (2016) further suggested that economic constraints limit capital resources that can be applied in the workplace, making it more difficult for individuals in economic constraints to acquire the resources that privileged individuals take for granted [17]. A study among labour market entrants found that individuals whose parents had higher education levels, were employed, or were from the service class (indicating greater economic income) had greater access to job-finding resources [41]. In other words, less economic constraint promotes greater access to job resources.

Based on these studies, we establish the second hypothesis：

**H2:** Work resources mediate the relationship between economic constraints and work volition.

**Family support and Work resources**

About the relationship between these two mediated variables, some studies indicated the relationship between these two mediating variables has been explored in several studies, which suggest that family support is associated with work resources. Several theories have demonstrated the relationship between family and work resources. Bourdieu's theory (2008) postulates that families with diverse capital can provide individuals with resources, including work-related resources such as a professional network, job opportunities, and a clear understanding of job rules [40]. Conservation of Resources theory raised by Hobfoll indicates that in order to prevent the loss of resources, individuals invest in resources and acquire the corresponding resources[63]. For college students, families make many investments in resources for their work. The family investment model suggests that families of higher socioeconomic status tend to leverage their financial and networking strengths for investment, which means more family support promotes more work resources[64]. Researchers have also conducted empirical studies to test this relationship. Fass et al. (2013) found that families with high occupational status can provide their children with work resources such as professional training, communication skills, and managerial abilities [43]. In other words, individuals with various forms of family support are likely to have access to ample work resources. Wayne and Matthews et al. (2020) conducted a cross-sectional survey of employees and found that work resources are associated with lower work-to-family conflict[44], which suggests that individuals can receive greater family support for their work. Furthermore, a study focusing on college students revealed that higher levels of family cohesion were associated with career thoughts, an important aspect of work volition. This suggests that a harmonious family can provide greater support and promote a higher level of work volition[45].

Based on these studies and combine the findings that family support and work resource play important roles between economic constraints and work volition, we established the third hypothesis:

H3: Family support and work resources played a chain-mediating role in the relationship between economic constraints and work volition.

**Reviewer: 2**

1. **I found some errors in your citations.**

**AUTHORS’ RESPONSE: Thank you very much for your review of our manuscript and very helpful comments. We have revised the citation format of 30 references and added 4 references.** **(pp. 17-23, marked in blue):**

**References**

1. Du Yang. The Trend of Changes in Employment of College Students and Suggestions for Countermeasures. People's Tribune. 2022;(17):96-98.
2. Duffy, R. D., Kim, H. J., Boren, S., Pendleton, L., & Perez, G. Lifetime experiences of economic constraints and marginalization among incoming college students: A latent profile analysis. Journal of Diversity in Higher Education. 2023;16(3), 384–396. <https://doi.org/10.1037/dhe0000344>
3. Wei, J., Chan, S. H. J., & Autin, K. Assessing perceived future decent work securement among Chinese impoverished college students. Journal of Career Assessment. 2022;30(1), 3–22. <https://doi.org/10.1177/10690727211005653.>
4. Duffy, R. D., Diemer, M. A., Perry, J. C., Laurenzi, C., & Torrey, C. L. The construction and initial validation of the work volition scale. Journal of Vocational Behavior. 2011; 80(2): 400–411. doi:10.1016/j.jvb.2011.04. 002
5. ZHANG Juncheng; HUANG Yingjie; ZHANG Shuying; LIU Dege; QU Shuguang.The Measurement and Individual Differences of College Students’ Work Volition. Psychology:Techniques and Applications. 2019;7(10):629-640.doi:10.16842/j.cnki.issn2095-5588.2019.10.008.
6. Allan, BA., Sterling, HM., Duffy, R.D. Longitudinal relations among economic deprivation, work volition, and academic satisfaction: a psychology of working perspective. International Journal for Educational and Vocational Guidance. 2020; 20:311–329. [https://doi.org/10.1007/s10775-019-09405-3.](https://doi.org/10.1007/s10775-019-09405-3。)
7. Kim, T., & Allan, B. A. Examining Classism and Critical Consciousness Within Psychology of Working Theory. Journal of Career Assessment. 2021; 29(4):644–660. <https://doi.org/10.1177/1069072721998418>
8. Wang, Z. Y. and C. K. Li, et al. Family Economic Strain and Adolescent Aggression during the COVID-19 Pandemic: Roles of Interparental Conflict and Parent-Child Conflict. APPLIED RESEARCH IN QUALITY OF LIFE. 2022;17(4): 2369-2385. doi:10.1007/s11482-022-10042-2
9. Sprung, J. M. Economic Stress, Family Distress, and Work-Family Conflict among Farm Couples. JOURNAL OF AGROMEDICINE. 2022;27(2): 154-168. doi:10.1080/1059924X.2021.1944417
10. Conger, R. D., Wallace, L. E., Sun, Y., Simons, R. L., Mcloyd, V. C., & Brody, G. H. Economic pressure in african american families: a replication and extension of the family stress model. Dev Psychol. 2002; 38(2): 179–193.
11. Newman, A. and K. Dunwoodie, et al. Openness to Experience and the Career Adaptability of Refugees: How Do Career Optimism and Family Social Support Matter? JOURNAL OF CAREER ASSESSMENT. 2022;30(2): 309-328. doi: 10.1177/10690727211041532
12. Agrawal, S. and S. Singh. Predictors of subjective career success amongst women employees: moderating role of perceived organizational support and marital status. GENDER IN MANAGEMENT. 2022;37(3): 344-359. DOI10.1108/GM-06-2020-0187
13. Mehreen, A. and Z. Ali. Really shocks can't be ignored: the effects of career shocks on career development and how family support moderates this relationship? 2022;INTERNATIONAL JOURNAL FOR EDUCATIONAL AND VOCATIONAL GUIDANCE. doi:10.1007/s10775-022-09574-8
14. Yang, D. L. and G. X. Fang, et al. Impact of work-family support on job burnout among primary health workers and the mediating role of career identity: A cross-sectional study. FRONTIERS IN PUBLIC HEALTH 11. 2023. doi:10.3389/fpubh.2023.1115792
15. Fouad, N. A. , Cotter, E. W. , Fitzpatrick, M. E. , Kantamneni, N. , Carter, L. , & Bernfeld, S. Development and validation of the family influence scale. Journal of Career Assessment. 2010；18(3), 276-291.
16. Pursio, H. and A. Siukola, et al. Associations between Work Resources and Work Ability among Forestry Professionals. SUSTAINABILITY. 2021;13(9). doi:10.3390/su13094822
17. Duffy, R. D., Blustein, D. L., Diemer, M. A., and Autin, K. L. The psychology of working theory. J. Counsel. Psychol. 2016; 63:127–148. <https://doi.org/10.1037/cou0000140>
18. Hai, L., Bao, X., & Li, W. Factors associated with work volition among Chinese undergraduates. Front. Psychol. 2022; 13:1037185. doi:10.3389/fpsyg. 2022.1037185
19. Zhao, F., Wang, Y. & Ping, L. Work volition and academic satisfaction in Chinese female college students: the moderating role of incremental theory of work volition. Int J Educ Vocat Guidance. 2022. <https://doi.org/10.1007/s10775-022-09542-2>
20. Kim, N.R., Kim, H.J., & Lee, K.H. Social Support and Occupational Engagement Among Korean Undergraduates: The Moderating and Mediating Effect of Work Volition. Journal of Career Development. 2018; 45:285–298.
21. Kim, N.R., Kim, H. J., & Lee, K.H. Social status and decent work: test of a moderated mediation model. The Career Development Quarterly. 2020; 68(3):208–221. <https://doi.org/10.1002/cdq.12232>
22. Brief, A. P., Konovsky, M. A., Goodwin, R., & Link, K. Inferring the	meaning of work from the effects of unemployment. Journal of Applied Social Psychology. 1995; 25(8): 693–711. https://doi. org/10.1111/j.1559-1816. 1995.tb01769.x
23. Duffy, R. D., Douglass, R. P., Autin, K. L., & Allan, B. A. . Examining predictors of work volition among undergraduate students. Journal of Career Assessment. 2016; 24(3), 441–459. doi:10.1177/1069072715599377
24. Cheung, F., Ngo, H. Y., & Leung, A. Predicting work volition among undergraduate students in the united states and Hong Kong. Journal of Career Development. 2020; 47(5): 565–578. <https://doi.org/10.1177/0894845318803469>
25. Tokar, D. M., & Kaut, K. P. Predictors of decent work among workers with Chiari malformation: an empirical test of the psychology of working theory. Journal of Vocational Behavior. 2018; 106(JUN.), 126–137.
26. Autin, K. L., Douglass, R. P., Duffy, R. D., England, J. W., & Allan, B. A. Subjective social status, work volition, and career adaptability: a longitudinal study. Journal of Vocational Behavior. 2017; 99:1–10. <https://doi.org/10.1016/j.jvb.2016.11.007>
27. Betz, N. E. Implications of the null environment hypothesis for women’s career development and for counseling psychology. The Counseling Psychologist. 1989; 17, 136–144. doi:10.1177/0011000089171008
28. Nota, L., Ferrari, L., Solberg, V. S. H., & Soresi, S. Career search self-efficacy, family support, and career indecision with Italian youth. Journal of Career Assessment. 2007; 15(2), 181–193.
29. Zeng, Q. and J. Li, et al. How does career-related parental support enhance career adaptability: the multiple mediating roles of resilience and hope. CURRENT PSYCHOLOGY. 2023;42(29): 25193-25205. doi:10.1007/s12144-022-03478-0
30. Zhang, J. H. and M. Yuen, et al. Career-Related Parental Support, Vocational Identity, and Career Adaptability: Interrelationships and Gender Differences. CAREER DEVELOPMENT QUARTERLY. 2021;69(2): 130-144. doi:10.1002/cdq.12254
31. Song, Y. and F. Mu, et al. The Relationships Between Career-Related Emotional Support From Parents and Teachers and Career Adaptability. FRONTIERS IN PSYCHOLOGY 13. 2022.
32. Conrad, A. and K. W. Paschall, et al. Persistent economic insecurity and harsh parenting: A latent transition analysis. CHILDREN AND YOUTH SERVICES REVIEW. 2019:101: 12-22. doi:10.1016/j.childyouth.2019.03.036.
33. Nuri, R. P., Batorowicz, B., & Aldersey, H. M. . Family support and family and child-related outcomes among families impacted by disability in low- and middle-income countries: a scoping review. Journal of Child Health Care, 2019; 24(4), 637–654. <https://doi.org/10.1177/1367493519883456>
34. Bakker, A. B. and Demerouti, E. The Job Demands‐Resources model: state of the art. Journal of Managerial Psychology. 2007; 22(3):309–328. <https://doi.org/10.1108/02683940710733115>
35. Janse van Rensburg, C., Rothmann, S., and Diedericks, E. Job demands and resources: flourishing and job performance in SouthAfrican universities of technology settings. J. Psychol. Africa. 2018;28, 291–297. doi: 10.1080/14330237.2018.1501881
36. Barbier, M., Hansez, I., Chmiel, N. & Demerouti, E. Performance expectations, personal resources, and job resources: How do they predict work engagement? European Journal of Work and Organizational Psychology. 2013; 22(6): 750–762. doi: 10.1080/1359432X.2012.704675
37. Takawira, N., Coetzee, M., and Schreuder, D. Job embeddedness, work engagement and turnover intention of staff in a higher education institution: an exploratory study. SA J. Hum. Resour. Manag. 2014;12, 1–10. doi: 10.4102/sajhrm.v12i1.524
38. Choi, N. Y. , Kim, Y. H. , & Evans, C. A. An examination of the psychology of working theory with employed asian american women:. The Counseling Psychologist. 2022;50(8), 1074-1095.
39. Zhang Juncheng, Huang Yingjie, Zhang Shuying et al. The Measurement and Individual Differences of College Students' Work Volition. Psychology:Techniques and Applications. 2019;7(10):629-640. DOI:10.16842/j.cnki.issn2095-5588.2019.10.008.
40. Bourdieu, P. The Forms of Capital. John Wiley & Sons, Ltd. 2008.
41. Verhaeghe, P. P. and Y. J. Li, et al. Socio-economic and Ethnic Inequalities in Social Capital from the Family among Labour Market Entrants.EUROPEAN SOCIOLOGICAL REVIEW. 2013;29(4): 683-694.444
42. Liu Xiaolin, Diao Jiaxi, Liu Yang et al. Analysis of Influencing Factors and Training Strategies of Adolescents' Anti-pressure Ability in the New Era. Shanxi Youth,2022(11):187-189.
43. Faas, C., Benson, M. J., & Kaestle, C. E. .Parent resources during adolescence: effects on education and careers in young adulthood. Journal of Youth Studies. 2013; 16(2): 151–171.
44. Wayne, J. H. and R. Matthews, et al. Predictors and processes of satisfaction with work-family balance: Examining the role of personal, work, and family resources and conflict and enrichment.HUMAN RESOURCE MANAGEMENT. 2020;59(1): 25-42.
45. Fornell, C.; Larcker, D. Evaluating structural equation models with unobservable variables and measurement error. J. Mark. Res. 1981;18, 39–50.
46. Henseler, J., C. M. Ringle & M. Sarstedt.. A new criterion for assessing discriminant validity in variance-based structural equation modeling [J]. Journal of the Academy of Marketing Science, 2015, 43(1): 115-135.]
47. Lustig, D. and Y. H. Xu. Family-of-Origin Influence on Career Thoughts. CAREER DEVELOPMENT QUARTERLY. 2018;66(2): 149-16
48. Hobfoll, S. E. Conservation of resources: a new attempt at conceptualizing stress. American Psychologist. 1989; 44(3): 513–524. https://doi.org/10.1037/0003-066X.44.3.513
49. Zimet, G. D., Dahlem, N. W., Zimet, S. G., & Farley, G. K. The multidimensional scale of perceived social support. Journal of Personality Assessment. 1988; 52(1): 30–41. doi:10.1207/s15327752jpa5201_2
50. Spreitzer, G. M. Social structural characteristics of psychological empowerment. Academy of Management Journal. 1996; 39(2): 483–504. doi:10.2307/256789
51. Duffy, R. D., Gensmer, N., Allan, B. A., Kim, H. J., Douglass, R. P., England, J. W., Blustein, D. L. Developing, validating, and testing improved measures within the psychology of working theory. Journal of Vocational Behavior. 2019; 112, 199–215. doi:10.1016/j.jvb.2019.02.012
52. Zimet, G. D., Dahlem, N. W., Zimet, S. G., & Farley, G. K. The multidimensional scale of perceived social support. Journal of Personality Assessment. 1988; 52(1): 30–41. doi:10.1207/s15327752jpa5201_2
53. Spreitzer, G. M. Social structural characteristics of psychological empowerment. Academy of Management Journal. 1996; 39(2): 483–504. doi:10.2307/256789
54. Duffy, Ryan, & D.Perceiving a calling, living a calling, and job satisfaction: testing a moderated, multiple mediator model. Journal of Counseling Psychology. 2012.
55. Weston, R., & Gore, P. A., Jr. A brief guide to structural equation modeling. The Counseling Psychologist, 2006; 34(5): 719–751. doi:10.1177/0011000006286345
56. Hair, J. F., Anderson, R. E., Tatham, R. L., & Black, W. C. Multivariate data analysis. Prentice Hall. 1998.
57. Fornell, C.; Larcker, D. Evaluating structural equation models with unobservable variables and measurement error. J. Mark. Res. 1981;18, 39–50.
58. Henseler, J., C. M. Ringle & M. Sarstedt.. A new criterion for assessing discriminant validity in variance-based structural equation modeling. Journal of the Academy of Marketing Science, 2015;43(1): 115-135.
59. Hobfoll, S. E. Conservation of resources: a new attempt at conceptualizing stress. American Psychologist. 1989; 44(3): 513–524. <https://doi.org/10.1037/0003-066X.44.3.513>
60. Lu Yuanyuan. Analysis on the value and risk of Personal network Resource Management in the workplace. Fortune Today, 2021;(18):211-213.
61. Wang,Y., Zhang Z.,&He Y. The Research on the Effects of Work-Family Support on Employees' Creativity. Acta Psychologica Sinica. 2012;44(12):1651-1662.
62. Melamed S, Shirom A, Toker S, et al, Burnout and risk of cardiovascular disease:evidence, possible causal paths, and promising research directions[J]. Psychologicalbulletin,2006;132(3): 327.
63. Hobfoll S E. The influence of culture, community, and the nested - self in the stressprocess: Advancing conservation of resources theory. Applied psychology. 2001;50(3): 337-421.

64.Conger,R.D.&M.B.Donnellan.An interactionist Perspective on the Socioeconomic Context of Human Development. Annual Review of Psychology. 2007;5.

2. **I found that in figure 1 there are two** **diagrams which are seemed to be similar.**

**AUTHORS’ RESPONSE: Thank you very much for your review of our manuscript and very helpful comments. The two diagrams are exactly the same, so we have deleted one.**

3. **AVE of work volition is less than .50. You might go for item deletion to make it above .50 as it seems a serious threat to convergent validity.**

**AUTHORS’ RESPONSE: Thank you very much for your review of our manuscript and very helpful comments. We have reprocessed the data. In this study, after removing two items based on the results, there are five items remaining, the internal consistency coefficient of the scale was α=0.84 and the AVE of work volition is .52, which is more than .50. ((pp. 10-11;15-17, marked in blue):**

**Results**

**Preliminary Analyses**

Before analyzing the data, we conducted preliminary analyses using IBM SPSS 21.0. The skewness and kurtosis of all variables were between -0.66 and 0.27 and between −0.78 and 0.33, respectively. On account of the absolute values of skewness ≤ 3.0 and kurtosis ≤ 10.0[55], the shape of the data distribution in the current study may not be severely non-normal. All items in the questionnaire were subjected to Harman’s single-factor test, and the results showed that the first factor explained 36.11% of the total variance, which is below the critical value of 40%. This indicates that there is no significant presence of common method bias.

Moreover, the results showed that the CR of the four scales were 0.95, 0.88, 0.90 and 0.84 respectively, denoting high composite reliability based on the principle of Hair et al.(1998)[56]. The results showed that the AVE of the four scales were 0.79, 0.65, 0.75 and 0.52, and the square root of the number AVE on the diagonal of the scale mostly higher than the Pearson correlation among scales, indicating that these questionnaire have strong convergent validity and discriminant validity based on the principle of Fornell and Larcker（1981）[45]. And, the results showed that the HTMT between the four scales were less than 0.85, further denoting high composite reliability based on the principle of Henseler, Ringle, & Sarstedt(2015)[58]. The descriptive statistics also are presented (see Table 1).

**Measurement Model**

We constructed measurement models to examine correlations among latent variables and the goodness of fit of the observed indicators on their associated latent constructs with Amos Graphics 24.0; we found χ^2^/df=4.78, RMSEA = 0.06, TLI = 0.97, CFI = 0.97, and SRMR = 0.04, indicating a good fit according to some thresholds^[1]^. We, then, tested these structural models. The factor correlations and descriptive statistics are presented in Table 1.

**Structural Model**

We tested structural models that contained all the hypothesized direct and indirect effects and found that most paths were significant. The results of *χ*^2^/df=4.78, RMSEA = 0.06, TLI = 0.97, CFI = 0.97, and SRMR = 0.04 indicate a good fit according to some thresholds^[2]^. In terms of direct paths, economic constraints failed to predict work volition directly (β = -0.05, SE = 0.03, p=0.13, 95% CI [-0.11, 0.01]). Economic constraints had significant direct effects on family support (β = -0.21, SE = 0.03, p <0.001, 95% CI [ -0.27, -0.14]) and work resources (β = -0.11, SE = 0.03, p <0.01, 95% CI [-0.18, -0.05]); family support had significant direct effects on work resources (β = 0.47, SE = 0.03, p <0.001, 95% CI [0.40, 0.53]) and work volition (β = 0.15, SE = 0.04, p <0.001, 95% CI [0.07, 0.23]); work resources had significant direct effects on work volition (β = 0.53, SE = 0.04, p <0.001, 95% CI [0.46, 0.60]).

As for the indirect paths, economic constraints had a significant indirect effect on work volition via family support and work resources (β = -0.02, SE = 0.01, p <0.001, 95% CI [-0.03, -0.01] and β = -0.04, SE = 0.01, p <0.01, 95% CI [-0.06, -0.02], respectively). Additionally, we examined a chain mediation that economic constraints to work volition via family support and work resources (β = -0.03, SE = 0.01, p <0.001, 95% CI [-0.05, -0.02]). All paths are listed in Tables 2 and 3.

**Table 1 Descriptive Statistics and Correlations of Variables**

|  | CR | AVE | 1 | 2 | 3 | 4 |
| --- | --- | --- | --- | --- | --- | --- |
| 1.Economic constraints | 0.95 | 0.79 | 0.89 | 0.24 | 0.22 | 0.20 |
| 2. Family Support | 0.88 | 0.65 | -0.22** | 0.81 | 0.51 | 0.45 |
| 3. Work Resources | 0.90 | 0.75 | -0.21** | 0.45** | 0.87 | 0.63 |
| 4. Work Volition | 0.84 | 0.52 | -0.18** | 0.38** | 0.55** | 0.72 |
| ***M*** | | | 3.78 | 5.14 | 3.50 | 4.84 |
| ***SD*** | | | 1.50 | 1.15 | 0.76 | 1.01 |

Note: ^*^*p* < 0.05, ^**^*p* < 0.01, ^***^*p* < 0.001. The data on the diagonal of different questionnaires represent the square root of AVE. The data below the diagonal represent the correlation coefficients between different questionnaires. The data above the diagonal represent the HTMT values of the questionnaires.

**Table 2 Paths Coefficient from Structural Model**

| **Paths** | **β** | **SE** | **95% confidence interval**  **bootstrap bias corrected** | |
| --- | --- | --- | --- | --- |
|  |  |  | **Lower bound** | **Upper bound** |
| EC→FS | -0.21^***^ | 0.03 | -0.27 | -0.14 |
| EC→WR | -0.11^**^ | 0.03 | -0.18 | -0.05 |
| EC→WV | -0.05 | 0.03 | -0.11 | 0.01 |
| FS→WR | 0.47^***^ | 0.03 | 0.40 | 0.53 |
| FS→WV | 0.15^***^ | 0.04 | 0.07 | 0.23 |
| WR→WV | 0.53^***^ | 0.04 | 0.46 | 0.60 |

Note: EC=economic constraints; FS=family support; WR=work resources; WV=work volition. ^*^*p* < 0.05, ^**^*p* < 0.01, ^***^*p* < 0.001

**Table 3 Direct and Indirect Effects from the Structural Model**

| **paths** | **effect type** | **β** | **SE** | **95% confidence interval bootstrap bias corrected** | | **ratio** |
| --- | --- | --- | --- | --- | --- | --- |
|  |  |  |  | **Lower bound** | **Upper bound** |  |
| EC→FS/WR  /FS&WR→WV | Direct effect | -0.03 | 0.02 | -0.07 | 0.01 | 0.60/0.44  /0.50 |
| EC→FS→WV | Total effcet | -0.05^*^ | 0.02 | -0.09 | -0.01 | - |
|  | Indirect effcet | -0.02^***^ | 0.01 | -0.03 | -0.01 | 0.40 |
| EC→WR→WV | Total effcet | -0.06^**^ | 0.01 | -0.11 | -0.02 | - |
|  | Indirect effcet | -0.04^**^ | 0.01 | -0.06 | -0.02 | 0.56 |
| EC→FS→WR→WV | Total effcet | -0.06^*^ | 0.02 | -0.10 | -0.02 | - |
|  | Indirect effcet | -0.03^***^ | 0.01 | -0.05 | -0.02 | 0.50 |

Note: EC=economic constraints; FS=family support; WR=work resources; WV=work volition. ^*^*p* < 0.05, ^**^*p* < 0.01, ^***^*p* < 0.001
