# Supplementary material for: Effect of family support and work resources in the relationship of economic constraints and work volition: Evidence from China
Source: PLoS One. 2025 Jan 17;20(1):e0310374. doi: 10.1371/journal.pone.0310374 (PMC11741595; doi:10.1371/journal.pone.0310374)
Supplement: Attachment — Submitted filename: Response to Reviewers.docx [file pone.0310374.s004.docx]

PONE-D-23-23939

Effect of Family Support and Work Resources in the Relationship of Economic Constraints and Work Volition : Evidence from China

PLOS ONE

Dear Dr. Henri Tilga

Thank you very much for the opportunity to revise our manuscript entitled “Effect of Family Support and Work Resources in the Relationship of Economic Constraint and Work Volition : Evidence from China”. We would like to thank you and the reviewers for their helpful comments. The manuscript has been carefully revised according to the comments raised by the reviewers. Please find a point-to-point response to reviewers’ comments below. Changes to the manuscript are highlighted in blue.

We believe that the revisions prompted by the reviewers’ comments have strengthened our manuscript. We hope it is now suitable for publication and look forward to hearing from you in course.

Yours faithfully

Reviewers’ Comments to Author:

**Reviewer: 1**

**1.The introductory section of the document lacks the requisite depth and detail. It is essential to provide a more comprehensive introduction, one that not only sets the stage for the study but also explicitly identifies the existing knowledge gaps that this research aims to address. Without a clear articulation of these gaps in the introduction, the readers may find it challenging to understand the context and significance of the research. The literature review is weak. The authors must explain the main theories in the field of the study and identify the gaps in previous models. By doing so, researchers can develop a more compelling and theoretically sound model that addresses the shortcomings of earlier studies.**

**AUTHORS’ RESPONSE:** **Thank you very much for your review of our manuscript and very helpful comments. We have fleshed out the introduction, particularly enriched the literature review. Moreover, we have detailed the contributions and shortcomings of previous research****. Previous research has not fully considered the mediating role of objective factors in the relationships between economic constraints and work volition, such as family support and work resources. Therefore, it has not explored the internal mechanisms that economic constraints use to influence work volition through family support and work resources have not been fully explored.****(pp. 3-4, marked in blue):**

Work volition is defined as an individual's sense of agency or freedom to make a career choice [4], highlighting the potential of subjective belief to overcome external constraints. For contemporary Chinese college students, their work volition exhibits two key characteristics. Firstly, overall, the level of work volition is generally low. Secondly, individuals from low-income families possess lower work volition than those from high socioeconomic status families [5]. Notably, economic constraints can negatively impact work volition, a finding consistently supported by some researches in different areas. It was found that individuals with greater economic constraints tend to have lower levels of career decision-making among university students and employees [6][7]. Meanwhile, The PWT (Duffy et al., 2016) was developed to capture the work lives of all individuals. In PWT moedel, economic constraints and economic constraints are positioned as key predictors, in particular, economic constraints can objectively predict work volition. Additionally, economic constraints can also escalate conflicts and distress within families [8][9], for example, young individuals from families who are economically disadvantaged tend to encounter both internal and external issues [10], undermining adolescents' access to family support. Given the strong emphasis on family values in Chinese culture, family support also plays a crucial role in individuals' growth and career development. Family support can bring emotional encouragement and work support to individuals, so that they feel more confident when they are making choice about work[11][12][13][14][15]. Furthermore, studies suggest that economic constraints are also associated with work resources [15], for instance, Duffy et al. (2016) suggested that economic constraints limit capital resources that can be applied in the workplace, making it more difficult for individuals in economic constraints to acquire the resources that privileged individuals take for granted [16]. And work resources are recognised as a fundamental requirement for individuals in career development [17].

Previous studies have examined the relationships among economic constraints, family support, work resources and work volition. It was demonstrated the direct effects of economic constraints on work volition and the direct impact of family support and work resources on work volition. However, there are still knowledge gaps. Obviously previous research has not fully considered the mediating role of objective factors in the relationships between economic constraints and work volition, such as family support and work resources. Therefore, it has not explored the internal mechanisms that economic constraints use to influence work volition through family support and work resources have not been fully explored.

**2.The segment dedicated to the** **literature review and hypotheses development exhibits some weaknesses. To bolster the quality of this section, it is imperative to delve into previous theories and research within the field. This entails a thorough discussion of the existing body of knowledge, highlighting key theories, findings, and their implications. Furthermore,** **it is crucial to elucidate the novel contribution of this research and provide a comprehensive explanation of how the model underpinning the study was constructed. The absence of this information leaves the model without a solid foundation, undermining the credibility and robustness of the research.**

**AUTHORS’ RESPONSE: Thank you very much for your review of our manuscript and very helpful comments. We have strengthened literature review and introduced the relevant theory, such as the PWT put forward by Duffy, which proved that economic constraints can objectively predict work volition. And we have provided a comprehensive explanation of how the model underpinning the study was constructed.(pp.8 marked in blue):**

The aim of this study was to examine the mechanisms underlying the relationship between economic constrains and work volition through the family support and work resources using a mediation model. In this research, three variables are positioned as key predictors of work volition: (a) economic constraints and (b) family support and (c) work resource as variables that both predict work volition and explain the mediating role of family support and work resource. Specifically, (1) family support mediates the relationship between economic constraints and work volition; (2) Work resources mediate the relationship between economic constraints and work volition; (3) Family support and work resources played a chain-mediating role in the relationship between economic constraints and work volition. Based on these three hypothesis pathways, we constructed the model. (See Figure1).

3. **The summary or conclusion of the study appears to be overly succinct and leaves some key elements unaddressed. A comprehensive conclusion should not only summarize the key findings but also provide answers to the research questions posed at the outset of the study. By failing to address these questions in the conclusion, the research may be perceived as incomplete and lacking in closure. A more comprehensive summary and conclusion would help readers to** **better grasp the implications and significance of the study's findings.**

**AUTHORS’RESPONSE: Thank you very much for your review of our manuscript and very helpful comments. We have added the summary and conclusion to make it more helpful for readers to better grasp the research. (pp.2,15 marked in blue):**

**Abstract**

**Background**

As the pace of economic development slows, college students are facing an increasingly challenging employment landscape. For instance, the expansion of higher education has led to a swell in the number of job seekers, which has in turn intensified competition. Given the limited job opportunities, it's understandable that many college students are developing a pessimistic employment mindset. Therefore, it's crucial to explore how objective factors influence their work aspirations. But few studies have explored the role of mediating factors between the two, such as family and resource factors.Thus, this study examines the effects of family support and work resources between the relationship between economic constraints and work volition.

**Methods**

The study examined 1249 Chinese undergraduate students as participants ((714 men and 535 women; M_age_ = 19.32, SD = 1.50), using the questionnaire with the Wenjuanxing online survey tool. The questionnaire were collected between August, 2022 and December, 2022. SPSS 21.0 and AMOS 24.0 were used to conducted a comprehensive analysis of the collected data, and investigate the relationships among latent variables and assess the goodness of fit of the observed indicators on their associated latent constructs. Additionally, we evaluated all the hypothesized direct and indirect effects.

**Results**

The results showed the direct and indirect relationships among economic constraints, family support, work resources and work volition. Economic constraints can directly predict work volition. Moreover, economic constraints have a significant negative impact on work volition via two mediators: family support and work resources. On the one hand, economic constraints negatively affect work volition through family support and work resource separately. On the other hand, economic constraints negatively predict family support and work resource, thus negatively impact work volition.

**Contribution**

The current study has established the independent mediating and chain-mediated effects of family support and work resources on the relationship between economic constraints and work volition. This deeper understanding of internal mechanisms provides valuable insights that can inform strategies for enhancing individual's work volition, particularly from the perspectives of economic constraints, family support, and work volition.

**Conclusion**

The present study, which focused on Chinese college students, delved into the relationship between economic constraints and work volition, taking into account the mediating effects of family support and work resources. The findings confirmed the mediating roles and chain mediating roles of these factors in the relationship. Specifically, three indirect pathways were identified: family support mediates the relationship between economic constraints and work volition; work resources mediate the relationship between economic constraints and work volition; family support and work resources played a chain-mediating role in the relationship between economic constraints and work volition. While acknowledging several limitations, the study provides valuable directions for future research on the importance of family support and work resources in career development. It also underscores the need for schools, families, and society to recognize and act on these factors to promote positive career outcomes for students.

**Reviewer: 2**

1. **One little correction is still needed. I found that the** **citation issue still prevails. Please correct it.**

**AUTHORS’ RESPONSE: Thank you very much for your review of our manuscript and very helpful comments. We have corrected the citation issue in** **the text and endnotes. (pp. 3-23, marked in blue):**

**References**

1. Du Y. The Trend of Changes in Employment of College Students and Suggestions for Countermeasures. People's Tribune. 2022;17:96-98.
2. Duffy, R. D., Kim, H. J., Boren, S., Pendleton, L., & Perez, G. Lifetime experiences of economic constraints and marginalization among incoming college students: A latent profile analysis. Journal of Diversity in Higher Education. 2023;16(3),:384–396. <https://doi.org/10.1037/dhe0000344>
3. Wei, J., Chan, S. H. J., & Autin, K. Assessing perceived future decent work securement among Chinese impoverished college students. Journal of Career Assessment. 2022;30(1):3–22. <https://doi.org/10.1177/10690727211005653.>
4. Duffy, R. D., Diemer, M. A., Perry, J. C., Laurenzi, C., & Torrey, C. L. The construction and initial validation of the work volition scale. Journal of Vocational Behavior. 2011; 80(2): 400–411. doi:10.1016/j.jvb.2011.04. 002
5. Zhang J, Huang Y J, Zhang S Y, Liu D G;, Qu S G. The Measurement and Individual Differences of College Students’ Work Volition. Psychology:Techniques and Applications. 2019;7(10):629-640.doi:10.16842/j.cnki.issn2095-5588.2019.10.008.
6. Allan, BA., Sterling, HM., Duffy, R.D. Longitudinal relations among economic deprivation, work volition, and academic satisfaction: a psychology of working perspective. International Journal for Educational and Vocational Guidance. 2020; 20:311–329. [https://doi.org/10.1007/s10775-019-09405-3.](https://doi.org/10.1007/s10775-019-09405-3。)
7. Kim, T., & Allan, B. A. Examining Classism and Critical Consciousness Within Psychology of Working Theory. Journal of Career Assessment. 2021; 29(4):644–660. <https://doi.org/10.1177/1069072721998418>
8. Wang, Z. Y. and C. K. Li, et al. Family Economic Strain and Adolescent Aggression during the COVID-19 Pandemic: Roles of Interparental Conflict and Parent-Child Conflict. APPLIED RESEARCH IN QUALITY OF LIFE. 2022;17(4): 2369-2385. doi:10.1007/s11482-022-10042-2
9. Sprung, J. M. Economic Stress, Family Distress, and Work-Family Conflict among Farm Couples. JOURNAL OF AGROMEDICINE. 2022;27(2): 154-168. doi:10.1080/1059924X.2021.1944417
10. Newman, A. and K. Dunwoodie, et al. Openness to Experience and the Career Adaptability of Refugees: How Do Career Optimism and Family Social Support Matter? JOURNAL OF CAREER ASSESSMENT. 2022;30(2): 309-328. doi: 10.1177/10690727211041532
11. Agrawal, S. and S. Singh. Predictors of subjective career success amongst women employees: moderating role of perceived organizational support and marital status. GENDER IN MANAGEMENT. 2022;37(3): 344-359. DOI10.1108/GM-06-2020-0187
12. Mehreen, A. and Z. Ali. Really shocks can't be ignored: the effects of career shocks on career development and how family support moderates this relationship? 2022;INTERNATIONAL JOURNAL FOR EDUCATIONAL AND VOCATIONAL GUIDANCE. doi:10.1007/s10775-022-09574-8
13. Yang, D. L. and G. X. Fang, et al. Impact of work-family support on job burnout among primary health workers and the mediating role of career identity: A cross-sectional study. FRONTIERS IN PUBLIC HEALTH 11. 2023. doi:10.3389/fpubh.2023.1115792
14. Conger, R. D., Wallace, L. E., Sun, Y., Simons, R. L., Mcloyd, V. C., & Brody, G. H. Economic pressure in african american families: a replication and extension of the family stress model. Dev Psychol. 2002; 38(2): 179–193.
15. Fouad, N. A. , Cotter, E. W. , Fitzpatrick, M. E. , Kantamneni, N. , Carter, L. , & Bernfeld, S. Development and validation of the family influence scale. Journal of Career Assessment. 2010；18(3), 276-291.
16. Duffy, R. D., Blustein, D. L., Diemer, M. A., and Autin, K. L. The psychology of working theory. J. Counsel. Psychol. 2016; 63:127–148. <https://doi.org/10.1037/cou0000140>
17. Pursio, H. and A. Siukola, et al. Associations between Work Resources and Work Ability among Forestry Professionals. SUSTAINABILITY. 2021;13(9). doi:10.3390/su13094822
18. Hai, L., Bao, X., & Li, W. Factors associated with work volition among Chinese undergraduates. Front. Psychol. 2022; 13:1037185. doi:10.3389/fpsyg. 2022.1037185
19. Zhao, F., Wang, Y. & Ping, L. Work volition and academic satisfaction in Chinese female college students: the moderating role of incremental theory of work volition. Int J Educ Vocat Guidance. 2022. <https://doi.org/10.1007/s10775-022-09542-2>
20. Kim, N.R., Kim, H.J., & Lee, K.H. Social Support and Occupational Engagement Among Korean Undergraduates: The Moderating and Mediating Effect of Work Volition. Journal of Career Development. 2018; 45:285–298.
21. Kim, N.R., Kim, H. J., & Lee, K.H. Social status and decent work: test of a moderated mediation model. The Career Development Quarterly. 2020; 68(3):208–221. <https://doi.org/10.1002/cdq.12232>
22. Brief, A. P., Konovsky, M. A., Goodwin, R., & Link, K. Inferring the	meaning of work from the effects of unemployment. Journal of Applied Social Psychology. 1995; 25(8): 693–711. https://doi. org/10.1111/j.1559-1816. 1995.tb01769.x
23. Duffy, R. D., Douglass, R. P., Autin, K. L., & Allan, B. A. . Examining predictors of work volition among undergraduate students. Journal of Career Assessment. 2016; 24(3), 441–459. doi:10.1177/1069072715599377
24. Cheung, F., Ngo, H. Y., & Leung, A. Predicting work volition among undergraduate students in the united states and Hong Kong. Journal of Career Development. 2020; 47(5): 565–578. <https://doi.org/10.1177/0894845318803469>
25. Tokar, D. M., & Kaut, K. P. Predictors of decent work among workers with Chiari malformation: an empirical test of the psychology of working theory. Journal of Vocational Behavior. 2018; 106(JUN.), 126–137.
26. Betz, N. E. Implications of the null environment hypothesis for women’s career development and for counseling psychology. The Counseling Psychologist. 1989; 17, 136–144. doi:10.1177/0011000089171008
27. Nota, L., Ferrari, L., Solberg, V. S. H., & Soresi, S. Career search self-efficacy, family support, and career indecision with Italian youth. Journal of Career Assessment. 2007; 15(2), 181–193.
28. Wang,Y., Zhang Z.,&He Y. The Research on the Effects of Work-Family Support on Employees' Creativity. Acta Psychologica Sinica. 2012;44(12):1651-1662.
29. Zeng, Q. and J. Li, et al. How does career-related parental support enhance career adaptability: the multiple mediating roles of resilience and hope. CURRENT PSYCHOLOGY. 2023;42(29): 25193-25205. doi:10.1007/s12144-022-03478-0
30. Zhang, J. H. and M. Yuen, et al. Career-Related Parental Support, Vocational Identity, and Career Adaptability: Interrelationships and Gender Differences. CAREER DEVELOPMENT QUARTERLY. 2021;69(2): 130-144. doi:10.1002/cdq.12254
31. Song, Y. and F. Mu, et al. The Relationships Between Career-Related Emotional Support From Parents and Teachers and Career Adaptability. FRONTIERS IN PSYCHOLOGY 13. 2022.
32. Bakker, A. B. and Demerouti, E. The Job Demands‐Resources model: state of the art. Journal of Managerial Psychology. 2007; 22(3):309–328. <https://doi.org/10.1108/02683940710733115>
33. Janse van Rensburg, C., Rothmann, S., and Diedericks, E. Job demands and resources: flourishing and job performance in SouthAfrican universities of technology settings. J. Psychol. Africa. 2018;28, 291–297. doi: 10.1080/14330237.2018.1501881
34. Melamed S, Shirom A, Toker S, et al, Burnout and risk of cardiovascular disease:evidence, possible causal paths, and promising research directions[J]. Psychologicalbulletin,2006;132(3): 327.
35. Barbier, M., Hansez, I., Chmiel, N. & Demerouti, E. Performance expectations, personal resources, and job resources: How do they predict work engagement? European Journal of Work and Organizational Psychology. 2013; 22(6): 750–762. doi: 10.1080/1359432X.2012.704675
36. Takawira, N., Coetzee, M., and Schreuder, D. Job embeddedness, work engagement and turnover intention of staff in a higher education institution: an exploratory study. SA J. Hum. Resour. Manag. 2014;12, 1–10. doi: 10.4102/sajhrm.v12i1.524
37. Choi, N. Y. , Kim, Y. H. , & Evans, C. A. An examination of the psychology of working theory with employed asian american women:. The Counseling Psychologist. 2022;50(8), 1074-1095.
38. Zhang Juncheng, Huang Yingjie, Zhang Shuying et al. The Measurement and Individual Differences of College Students' Work Volition. Psychology:Techniques and Applications. 2019;7(10):629-640. DOI:10.16842/j.cnki.issn2095-5588.2019.10.008.
39. Bourdieu, P. The Forms of Capital. John Wiley & Sons, Ltd. 2008.
40. Verhaeghe, P. P. and Y. J. Li, et al. Socio-economic and Ethnic Inequalities in Social Capital from the Family among Labour Market Entrants.EUROPEAN SOCIOLOGICAL REVIEW. 2013;29(4): 683-694.444
41. Hobfoll S E. The influence of culture, community, and the nested - self in the stressprocess: Advancing conservation of resources theory. Applied psychology. 2001;50(3): 337-421.
42. Conger,R.D.&M.B.Donnellan.An interactionist Perspective on the Socioeconomic Context of Human Development. Annual Review of Psychology. 2007;5.
43. Faas, C., Benson, M. J., & Kaestle, C. E. .Parent resources during adolescence: effects on education and careers in young adulthood. Journal of Youth Studies. 2013; 16(2): 151–171.
44. Wayne, J. H. and R. Matthews, et al. Predictors and processes of satisfaction with work-family balance: Examining the role of personal, work, and family resources and conflict and enrichment.HUMAN RESOURCE MANAGEMENT. 2020;59(1): 25-42.
45. Fornell, C.; Larcker, D. Evaluating structural equation models with unobservable variables and measurement error. J. Mark. Res. 1981;18, 39–50.
46. Duffy, R. D., Gensmer, N., Allan, B. A., Kim, H. J., Douglass, R. P., England, J. W., Blustein, D. L. Developing, validating, and testing improved measures within the psychology of working theory. Journal of Vocational Behavior. 2019; 112, 199–215. doi:10.1016/j.jvb.2019.02.012
47. Zimet, G. D., Dahlem, N. W., Zimet, S. G., & Farley, G. K. The multidimensional scale of perceived social support. Journal of Personality Assessment. 1988; 52(1): 30–41. doi:10.1207/s15327752jpa5201_2
48. Spreitzer, G. M. Social structural characteristics of psychological empowerment. Academy of Management Journal. 1996; 39(2): 483–504. doi:10.2307/256789
49. Duffy, Ryan, & D.Perceiving a calling, living a calling, and job satisfaction: testing a moderated, multiple mediator model. Journal of Counseling Psychology. 2012.
50. Weston, R., & Gore, P. A., Jr. A brief guide to structural equation modeling. The Counseling Psychologist, 2006; 34(5): 719–751. doi:10.1177/0011000006286345
51. Hair, J. F., Anderson, R. E., Tatham, R. L., & Black, W. C. Multivariate data analysis. Prentice Hall. 1998.
52. Fornell, C.; Larcker, D. Evaluating structural equation models with unobservable variables and measurement error. J. Mark. Res. 1981;18, 39–50.
53. Henseler, J., C. M. Ringle & M. Sarstedt.. A new criterion for assessing discriminant validity in variance-based structural equation modeling. Journal of the Academy of Marketing Science, 2015;43(1): 115-135.
54. Lu Y Y. Analysis on the value and risk of Personal network Resource Management in the workplace. Fortune Today, 2021;(18):211-213.
55. Hobfoll, S. E. Conservation of resources: a new attempt at conceptualizing stress. American Psychologist. 1989; 44(3): 513–524. <https://doi.org/10.1037/0003-066X.44.3.513>
56. Lu Y Y. Analysis on the value and risk of Personal network Resource Management in the workplace. Fortune Today, 2021;(18):211-213.
